# Maternal and perinatal risks for monozygotic twins conceived following frozen-thawed embryo transfer: a retrospective cohort study

**DOI:** 10.1186/s13048-024-01349-9

**Published:** 2024-02-07

**Authors:** Jing Lin, Kai Zhang, Fenglu Wu, Bian Wang, Weiran Chai, Qianqian Zhu, Jialyu Huang, Jiaying Lin

**Affiliations:** 1 Center for Reproductive Medicine, Xinhua Hospital, Shanghai Jiao Tong University School of Medicine, Shanghai, 200092 China; 2 Department of Hepatobiliary Surgery, National Clinical Research Center for Cancer / Cancer Hospital, National Cancer Center, Chinese Academy of Medical Sciences (CAMS) and Peking Union Medical College (PUMC), Beijing, 100021 China; 3 Department of Assisted Reproduction, Shanghai Ninth People’s Hospital, Shanghai Jiao Tong University School of Medicine, Zhizaoju Road No. 639, Shanghai, 200011 China; 4 Center for Reproductive Medicine, Jiangxi Maternal and Child Health Hospital, Nanchang University School of Medicine, 318 Bayi Avenue, Nanchang, 330006 China

**Keywords:** Monozygotic twins, Dizygotic twins, Neonatal death, Maternal and neonatal outcomes, Frozen-thawed embryo transfer

## Abstract

**Background:**

The present study aimed to explore the maternal and perinatal risks in cases of monozygotic twins (MZT) following frozen-thawed embryo transfer (FET).

**Methods:**

All twin births that were conceived following FET from 2007 to 2021 at Shanghai Ninth People’s Hospital in Shanghai, China were retrospectively reviewed. The exposure variable was twin type (monozygotic and dizygotic). The primary outcome was the incidence of neonatal death while secondary outcomes included hypertensive disorders of pregnancy, gestational diabetes, intrahepatic cholestasis of pregnancy, placenta previa, placental abruption, preterm premature rupture of the membranes, Cesarean delivery, gestational age, birth weight, weight discordance, stillbirth, birth defects, pneumonia, respiratory distress syndrome, necrotizing enterocolitis, and neonatal jaundice. Analysis of the outcomes was performed using logistic regression models to estimate odds ratios (ORs) and 95% confidence intervals (CIs). The causal mediation analysis was conducted. A doubly robust estimation model was used to validate the results. Kaplan-Meier method was used to calculate survival probability. The sensitivity analysis was performed with a propensity score-based patient-matching model.

**Results:**

Of 6101 dizygotic twin (DZT) and 164 MZT births conceived by FET, MZT showed an increased risk of neonatal death based on the multivariate logistic regression models (partially adjusted OR: 4.19; 95% CI, 1.23–10.8; fully adjusted OR: 4.95; 95% CI, 1.41–13.2). Similar results were obtained with the doubly robust estimation. Comparing MZT with DZT, the neonatal survival probability was lower for MZT (*P* < 0.05). The results were robust in the sensitivity analysis. Females with MZT pregnancies exhibited an elevated risk of preterm premature rupture of the membranes (adjusted OR: 2.42; 95% CI, 1.54–3.70). MZT were also associated with higher odds of preterm birth (prior to 37 weeks) (adjusted OR: 2.31; 95% CI, 1.48–3.67), low birth weight (adjusted OR: 1.92; 95% CI, 1.27–2.93), and small for gestational age (adjusted OR: 2.18; 95% CI, 1.21–3.69) in the fully adjusted analyses. The effect of MZT on neonatal death was partially mediated by preterm birth and low birth weight (*P* < 0.05).

**Conclusions:**

This study indicates that MZT conceived by FET are related to an increased risk of neonatal death, emphasizing a potential need for comprehensive antenatal surveillance in these at-risk pregnancies.

## Background

Assisted reproductive technology (ART) has been associated with multiple gestations, which can be attributed to the transfer of two or more embryos during the procedure. Dizygotic twins (DZT), which originate from two fertilized oocytes, are typically characterized by dichorionic-diamniotic (DCDA) placentation [1]. On the other hand, monozygotic twins (MZT) arise from one single fertilized oocyte that divides, resulting in DCDA (20–30%), monochorionic-diamniotic (MCDA, 70–75%), or monochorionic-monoamniotic (MCMA, 1–2%) pregnancies, depending on the timing of embryo division [2, 3].

In comparison with a spontaneous conception rate of 0.4% of live births [4], MZT pregnancies resulting from ART range from 0.7 to 5.6% [5–10]. However, the actual incidence of MZT after ART often remains underestimated primarily because twin gestation of a DCDA placentation following a multi-embryo transfer is generally presumed to be DZT [11, 12]. The causes of the increased risk of MZT after ART remain unclear, but recent systematic reviews have identified the younger maternal age, blastocyst transfer, extended culture to blastocyst stage, and gonadotropin-releasing hormone (GnRH) agonist suppression protocol as potential risk factors [13, 14]. The occurrence of MZT poses a clinical concern due to the adverse outcomes associated with twin pregnancies in general and MZT in particular. There are severe complications associated with monochorionic placentation, including twin-to-twin transfusion syndrome (TTTS), twin reversed arterial perfusion sequence (TRAPS), twin anemia-polycythemia sequence (TAPS), single intrauterine fetal demise, and selective intrauterine growth restriction [15]. Miscarriage, preterm delivery, low birth weight, growth restriction, developmental anomalies, preeclampsia, as well as perinatal morbidity and mortality are all increased [16–19].

Most research regarding MZT in ART has mainly centered on determining its incidence and etiology, with limited attention given to the clinical outcomes of MZT relative to DZT. This is an issue of great clinical significance, as twin pregnancies are associated with increased risks of maternal and fetal complications that may be influenced by zygosity. To understand this clinically relevant argument, we conducted a retrospective cohort investigation with the aim of providing deeper insights into the clinical outcomes of MZT pregnancies.

## Materials and methods

### Study population and design

**Fig. 1:**
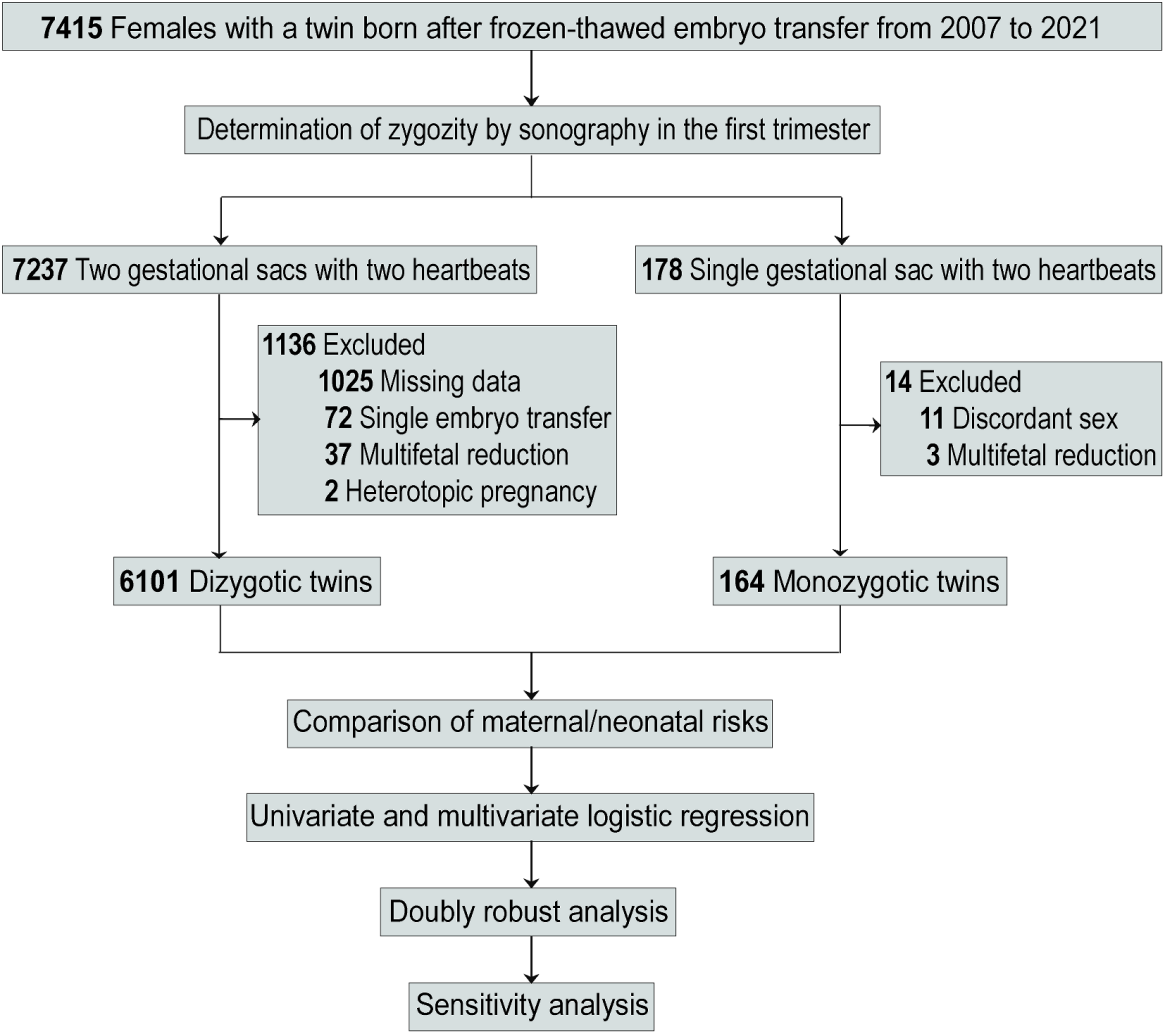
Flowchart of study participants

The study cohort selected for this analysis consisted of all females that gave birth to twins conceived via frozen-thawed embryo transfer (FET) between 2007 and 2021 at Shanghai Ninth People’s Hospital, Shanghai Jiao Tong University School of Medicine, Shanghai, China (*n* = 7415) (Fig. 1). The Institutional Review Board of Shanghai Ninth People’s Hospital approved this retrospective cohort study. Due to the deidentification of data and the retrospective nature of all analyses, the requirement for informed consent was waived.

The MZT was initially identified when two fetal poles were observed within one single intrauterine gestational sac in a transvaginal ultrasound performed between 6 and 7 weeks of gestation. A follow-up ultrasound was required to establish chorionicity, and pregnancies were accepted as monochorionic based on the absence of a ‘‘twin peak’’ sign (or lambda sign). Any women that had given birth to sex discordant newborns or experienced multifetal reduction were excluded from this study. Cases of monochorionic twin-specific complications such as TTTS, TRAPS, and TAPS were eliminated from this study.

The controls were women displaying DZT pregnancies, and the criteria for eligibility were: (1) evidence of two intrauterine gestational sacs, and (2) the presence of a ‘‘twin peak’’ sign in detailed ultrasound analysis. Those with a single embryo transfer, a multifetal reduction, a heterotopic pregnancy, or incomplete data were excluded.

### Outcome measures

Patient variables were extracted from medical records. The study’s objectives were to determine the maternal and neonatal risks associated with zygosity. The independent variable was twin type (monozygotic and dizygotic). The primary outcome variable in this study was the incidence of neonatal death, defined by mortality prior to hospital discharge after live birth. Other neonatal outcomes included gestational age (preterm birth [< 37 weeks], very preterm birth [< 32 weeks], extremely preterm birth [< 28 weeks]), birth weight (low birth weight [< 2500 g], very low birth weight [< 1500 g], macrosomia [> 4000 g], small for gestational age [SGA], large for gestational age [LGA]), birth weight discordance (defined by the percentage of intertwin birth weight difference ≥ 20%), stillbirth (defined by fetal death after a gestational age of 20 weeks and prior to or during delivery), birth defects (International Statistical Classification of Diseases and Related Health Problems—Tenth Revision [ICD-10 code]: Q00-Q99), pneumonia (P23), respiratory distress syndrome (P22), necrotizing enterocolitis (P77), and neonatal jaundice (P59). In this study, SGA and LGA were defined as birth weight values below the 10th percentile and above the 90th percentile, respectively, for Chinese twins of a particular gestational age [20, 21]. Maternal outcomes included hypertensive disorders of pregnancy (O13-O15), gestational diabetes (O24), intrahepatic cholestasis of pregnancy (O26), placenta previa (O44), placental abruption (O45), preterm premature rupture of the membranes (PPROM, O42), Cesarean delivery (O82).

### Statistical analyses

The normality of quantitative data distributions was analyzed with the Shapiro-Wilk test. A descriptive analysis of the patient’s characteristics was performed using mean and standard deviation (SD) for normally distributed quantitative data and counts and percentages for qualitative data. Between-group differences were evaluated by means of the Student’s *t* test for normally distributed quantitative data and Chi-square test for qualitative data.

We used three binomial logistic regression models with varying degrees of covariate adjustment to estimate the odds ratios (ORs) and corresponding 95% confidence intervals (CIs) for the analyzed outcomes. Firstly, a univariate analysis was conducted in Model 1 without adjusting for any covariates. Secondly, Model 2 was modified with twelve baseline demographic factors considered to be confounders by statistical analyses and clinical judgments. They were listed as follows: maternal age of delivery, infertility duration, gravidity, parity, pregestational body mass index (BMI), number of oocytes retrieved, fertilization method, FET cycle rank, FET protocol, stage of embryos transferred, number of embryos transferred, and year of treatment. Thirdly, Model 3 was modified with all twelve baseline covariates and seven maternal complications. A generalized variance inflation factor analysis was conducted to assess the multi-collinearity among the variables in our models. The interaction effects were examined by Friedman’s H-statistic. Furthermore, we conducted causal mediation analysis to elucidate the extent to which the effect of monozygotic twinning on neonatal death was mediated by intermediate variables.

To further validate the aforementioned findings, doubly robust estimation techniques were employed, with the same set of covariates as in Model 2 and Model 3. A weighted cohort was generated from the calculated propensity scores using an inverse probabilities of treatment weighting (IPTW) model, and then logistic regression was used to adjust for imperfect covariate balancing. The doubly robust estimator combines IPTW and outcome regression models to ensure consistency if either model is correct and efficiency if both are accurately specified. This approach provides two opportunities for obtaining a valid estimate of causal effects [22].

**Fig. 2:**
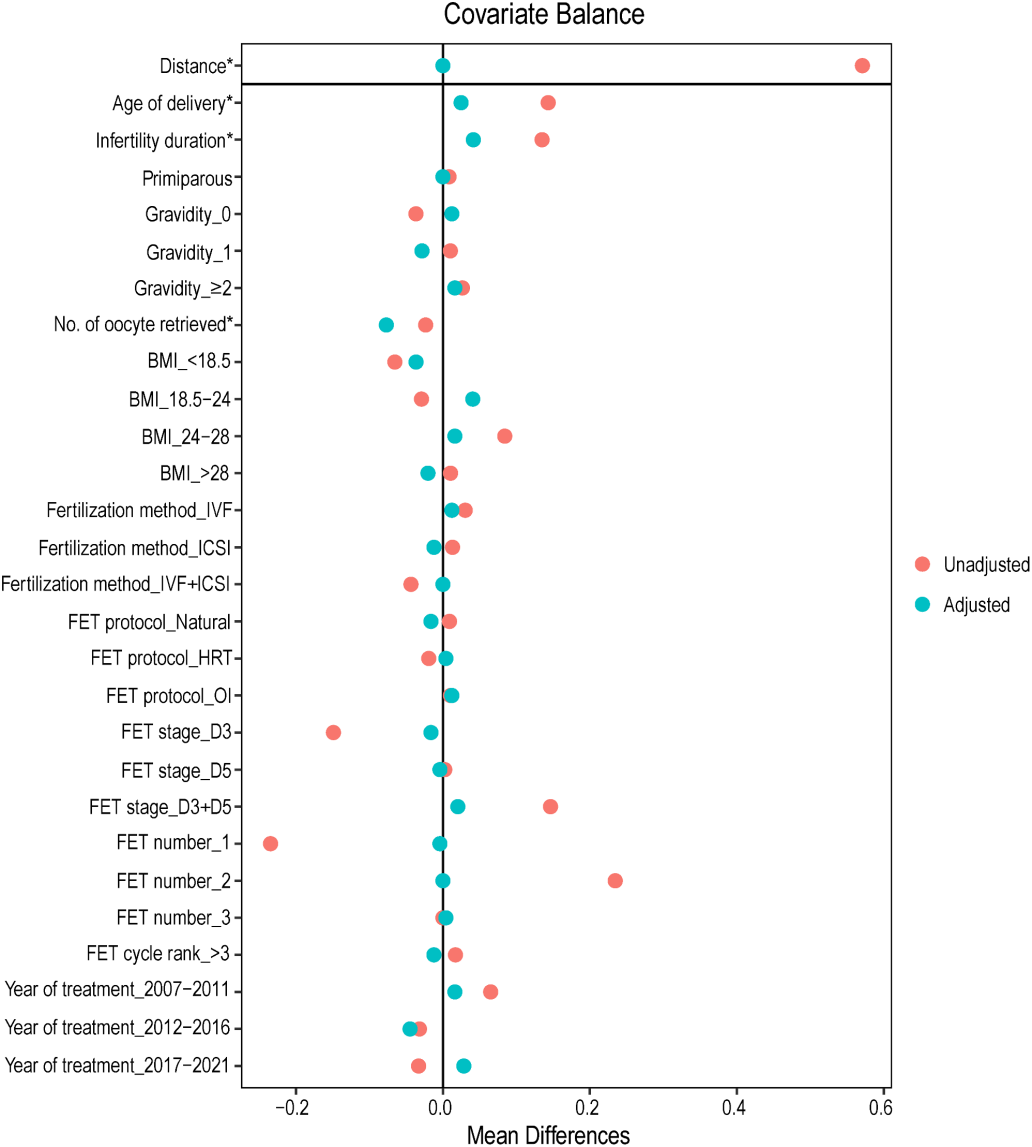
The comparison for imbalance of covariates in the original and the adjusted (weighted) cohorts in the propensity score-based patient-matching model. Mean differences are represented with red (unadjusted) and green dots (adjusted). Variables with standardized mean differences are marked with an asterisk. BMI, body mass index; FET, frozen embryo transfer; HRT, hormone replacement therapy; ICSI, intracytoplasmic sperm injection; IVF, in vitro fertilization; OI, ovulation induction

The sensitivity analysis was conducted using a propensity score-based patient-matching (PSM) model, a method that balances the distribution of baseline confounders across groups. Females with MZT and DZT pregnancies were matched (1:2) based on their estimated propensity scores derived from a binary logistic regression analysis, using the nearest neighbor method within a caliper of 0.05. The standardized mean difference (SMD) of effect sizes was calculated to account for the differences between the original and the adjusted cohorts; a covariate with a SMD less than 0.05 was considered adequately matched (Fig. 2). Kaplan-Meier curves were used to estimate the overall survival, and group differences were compared with a log-rank test.

The statistical analyses were conducted with the R software version 4.0.3, with a two-sided *P* < 0.05 as the significance cut-off. Data analyses were performed between February and August of 2023.

## Results

### Patient characteristics

The final study population consisted of 6101 DZT and 164 MZT births, as shown in Fig. 1. A comparison of the baseline characteristics is presented in Table 1. The distributions of pregestational BMI, stage of embryos transferred, number of embryos transferred, and year of treatment were found to be significantly different between the MZT and DZT groups (*P* < 0.05). However, other variables, including maternal age of delivery, maternal age of oocyte retrieval, duration of infertility, gravidity, primiparous status, number of oocytes retrieved, fertilization method, FET cycle rank, and FET protocol, were similar between the two groups (all *P* > 0.05).

**Table 1:** Characteristics of study participants

Characteristics	DZT (*N* = 6101)	MZT (*N* = 164)	P value
Maternal age of delivery, mean (SD), y	31.8 (3.69)	32.5 (4.13)	0.061
Maternal age of oocyte retrieval, mean (SD), y	30.6 (3.64)	31.1 (4.07)	0.121
Duration of infertility, mean (SD), y	3.04 (2.48)	3.43 (2.65)	0.067
Gravidity			0.591
0	3527 (57.8%)	89 (54.3%)	
1	1408 (23.1%)	39 (23.8%)	
≥2	1166 (19.1%)	36 (22.0%)	
Primiparous status	5661 (92.8%)	151 (92.1%)	0.845
Pregestational BMI, kg/m^2^			0.006
<18.5	646 (10.6%)	31 (18.9%)	
18.5–24	4193 (68.7%)	102 (62.2%)	
24–28	959 (15.7%)	21 (12.8%)	
>28	235 (3.85%)	8 (4.88%)	
Number of oocytes retrieved	13.2 (7.91)	13.1 (8.68)	0.881
Fertilization method			0.249
IVF	3778 (61.9%)	107 (65.2%)	
ICSI	1533 (25.1%)	43 (26.2%)	
IVF + ICSI	790 (12.9%)	14 (8.54%)	
FET cycle rank			0.524
1–3	5746 (94.2%)	152 (92.7%)	
4–11	355 (5.82%)	12 (7.32%)	
FET protocol			0.897
Natural	1296 (21.2%)	36 (22.0%)	
HRT	2036 (33.4%)	52 (31.7%)	
OI	2756 (45.2%)	76 (46.3%)	
Stage of embryos transferred			< 0.001
D2-3	5351 (87.7%)	119 (72.6%)	
D5-6	728 (11.9%)	44 (26.8%)	
Cleavage-stage embryo + blastocyst	22 (0.36%)	1 (0.61%)	
Number of embryos transferred			< 0.001
1	0 (0.00%)	38 (23.2%)	
2	6063 (99.4%)	125 (76.2%)	
3	38 (0.62%)	1 (0.61%)	
Year of treatment	0 (0.00%)	38 (23.2%)	< 0.001
2007–2011	214 (3.51%)	16 (9.76%)	
2012–2016	3403 (55.8%)	87 (53.0%)	
2017–2021	2484 (40.7%)	61 (37.2%)	

BMI, body mass index; DZT, dizygotic twins; FET, frozen embryo transfer; ICSI, intracytoplasmic sperm injection; IVF, in vitro fertilization; MZT, monozygotic twins; SD, standard deviation

### Primary outcome

As shown in Table 2, although the crude odds ratio (cOR) for neonatal death in MZT was not statistically significant (cOR 3.12; 95% CI, 0.94–7.78), the adjusted analyses revealed a significant difference (Model 2: adjusted odd ratio [aOR]: 4.19; 95% CI, 1.23–10.8; Model 3: aOR: 4.95; 95% CI, 1.41–13.2). Furthermore, based on the doubly robust estimation, the risk of neonatal death for MZT was significantly higher compared with DZT using variables in Model 2 (OR: 4.08; 95% CI, 1.40–11.9) or Model 3 (OR: 4.68; 95% CI, 1.66–13.2).

**Table 2:** Analysis of odds ratios of neonatal risks in monozygotic twins compared to dizygotic twins

Neonatal outcomes	DZT (*N* = 6101)	MZT (*N* = 164)	cOR (95% CI)	aOR (95% CI)^a^	aOR (95% CI)^b^
Neonatal death	49 (0.80%)	4 (2.47%)	3.12 (0.94–7.78)	4.19 (1.23–10.8)	4.95 (1.41–13.2)
Preterm birth, < 37 weeks	3043 (50.3%)	119 (75.3%)	3.01 (2.11–4.39)	2.55 (1.72–3.89)	2.31 (1.48–3.67)
Very preterm birth, < 32 weeks	241 (3.99%)	5 (3.16%)	0.79 (0.28–1.74)	0.85 (0.26–2.05)	0.87 (0.26–2.22)
Extremely preterm birth, < 28 weeks	40 (0.66%)	1 (0.63%)	0.96 (0.05–4.44)	1.38 (0.08–6.56)	1.80 (0.09-10.0)
Low birth weight, < 2500 g	3267 (54.0%)	111 (70.3%)	2.01 (1.43–2.86)	2.08 (1.41–3.14)	1.92 (1.27–2.93)
Very low birth weight, < 1500 g	216 (3.57%)	7 (4.43%)	1.25 (0.53–2.51)	1.23 (0.43–2.77)	1.20 (0.41–2.79)
Small for gestational age	376 (6.22%)	19 (12.0%)	2.06 (1.22–3.28)	2.20 (1.23–3.70)	2.18 (1.21–3.69)
Large for gestational age	1139 (18.8%)	19 (12.0%)	0.59 (0.35–0.93)	0.53 (0.28–0.91)	0.55 (0.29–0.95)
Macrosomia, > 4000 g	7 (0.12%)	0 (0.00%)	NA	NA	NA
Birth weight discordance	783 (12.9%)	15 (9.49%)	0.71 (0.40–1.17)	0.59 (0.29–1.08)	0.57 (0.28–1.04)
Stillbirth	5 (0.08%)	2 (1.22%)	15.1 (2.14–70.4)	10.7 (0.53–76.5)	12.0 (0.55–99.4)
Birth defects	188 (3.08%)	5 (3.09%)	1.00 (0.35–2.22)	1.52 (0.53–3.43)	1.39 (0.48–3.17)
Neonatal jaundice	115 (1.90%)	4 (2.53%)	1.34 (0.41–3.24)	1.96 (0.59–4.84)	1.92 (0.58–4.77)
Pneumonia	53 (0.88%)	2 (1.27%)	1.45 (0.24–4.72)	2.25 (0.36–7.51)	2.47 (0.39–8.32)
Respiratory distress syndrome	20 (0.33%)	0 (0.00%)	NA	NA	NA
Necrotizing enterocolitis	7 (0.12%)	0 (0.00%)	NA	NA	NA

aOR, adjusted odds ratio; CI, confidence interval; cOR, crude odds ratio; DZT, dizygotic twins; MZT, monozygotic twins; NA, not applicable

^a^Adjusted for maternal baseline characteristics

^b^Adjusted for maternal characteristics and maternal complications

**Fig. 3:**
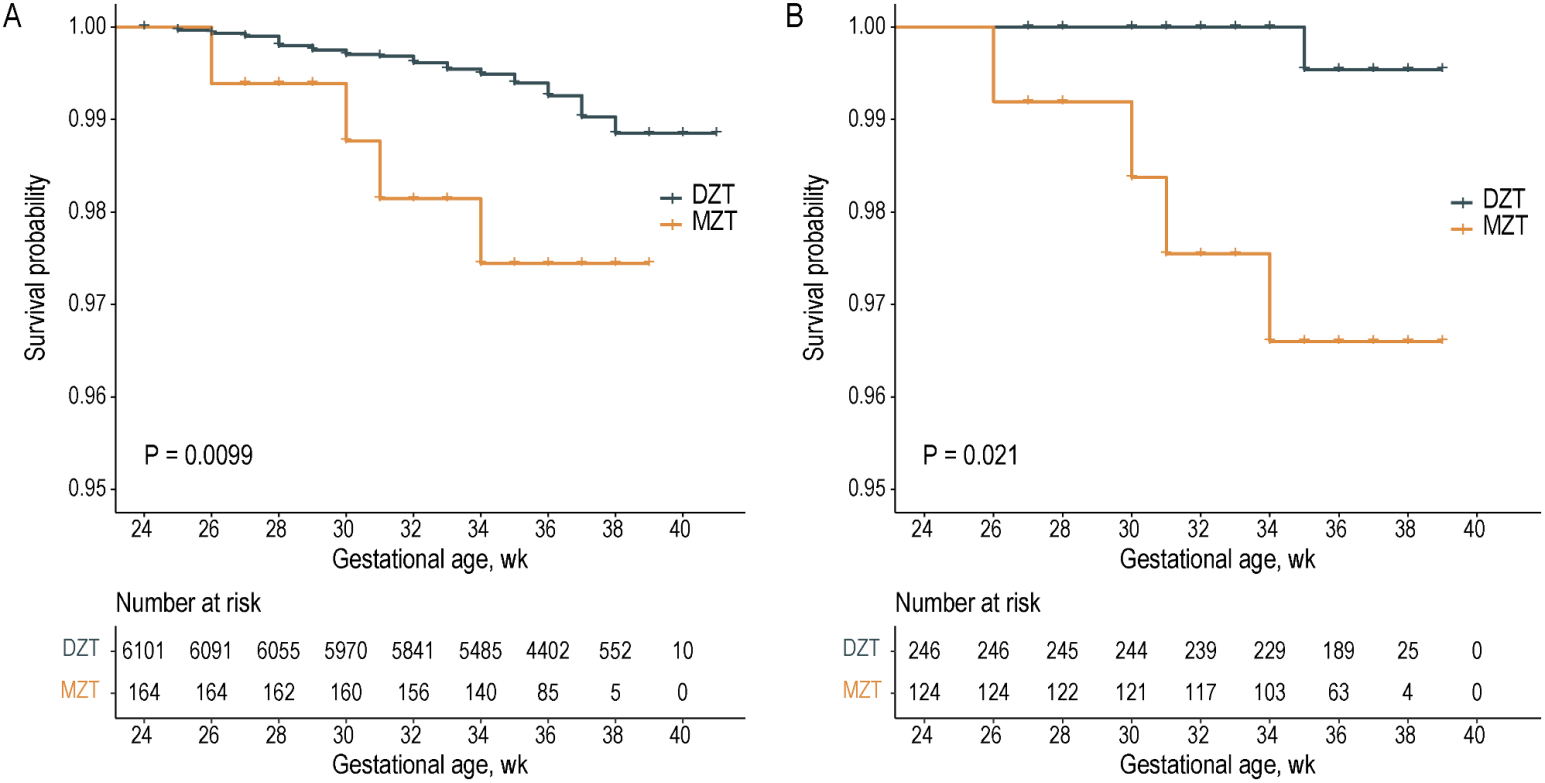
Analysis of neonatal mortality by Kaplan–Meier plots. A comparison of neonatal survival probability for MZT versus DZT before (**A**) and after (**B**) the propensity score-based patient-matching. DZT, dizygotic twins; MZT, monozygotic twins

In sensitivity analysis, 124 females with MZT were matched to 246 females with DZT following the 1-to-2 matching by propensity score, and the baseline covariates were well-balanced between the two groups as evidenced by small SMDs (Fig. 2). The sensitivity analysis yielded similar results (OR: 8.24; 95% CI, 1.20–162). Finally, Kaplan-Meier analysis of the pre- or post-PSM data demonstrated that MZT was associated with a lower neonatal survival probability (Fig. 3; both *P* < 0.05).

### Secondary outcomes

The neonatal outcomes are compared in Table 2. MZT were associated with higher odds of preterm birth (prior to 37 weeks) (aOR: 2.31; 95% CI, 1.48–3.67), low birth weight (aOR: 1.92; 95% CI, 1.27–2.93), and SGA (aOR: 2.18; 95% CI, 1.21–3.69) in the fully adjusted analyses. Compared to DZT, MZT had a lower risk of being born LGA (aOR: 0.55; 95% CI, 0.29–0.95). The results of the fully adjusted analyses were similar to those that were partially adjusted. Despite the significantly higher cOR for stillbirth in MZT (cOR 15.1; 95% CI, 2.14–70.4), the adjusted analyses did not reveal a statistically significant difference (aOR: 12.0; 95% CI, 0.55–99.4). The odds of very preterm birth, extremely preterm birth, very low birth weight, birth weight discordance, neonatal jaundice, pneumonia, and birth defects were not statistically different in the MZT versus DZT groups.

The maternal outcomes are presented in Table 3. Females with MZT pregnancies exhibited an increased risk of PPROM (aOR: 2.42; 95% CI, 1.54–3.70). There were no significant differences between MZT and DZT for several other maternal complications, including hypertensive disorders of pregnancy, gestational diabetes, intrahepatic cholestasis of pregnancy, placenta previa, and Cesarean delivery.

**Table 3:** Analysis of odds ratios of maternal risks in monozygotic twins compared to dizygotic twins

Maternal outcomes	DZT (*N* = 6101)	MZT (*N* = 164)	cOR (95% CI)	aOR (95% CI)^a^
Hypertensive disorders of pregnancy	471 (7.72%)	12 (7.32%)	0.94 (0.49–1.64)	0.78(0.32–1.58)
Gestational diabetes	564 (9.24%)	9 (5.49%)	0.57 (0.27–1.06)	0.66(0.28–1.35)
Intrahepatic cholestasis of pregnancy	58 (0.95%)	3 (1.83%)	1.94 (0.47–5.32)	1.99(0.32–6.68)
Placenta previa	47 (0.77%)	4 (2.44%)	3.22 (0.96–8.03)	1.95 (0.31–6.59)
Placental abruption	17 (0.28%)	0 (0.00%)	NA	NA
Preterm premature rupture of the membranes	925 (15.2%)	42 (25.6%)	1.93 (1.33–2.73)	2.42 (1.54–3.70)
Cesarean delivery	5874 (96.3%)	160 (97.6%)	1.55 (0.65–5.05)	1.17(0.48–3.85)

aOR, adjusted odds ratio; CI, confidence interval; cOR, crude odds ratio; DZT, dizygotic twins; MZT, monozygotic twins; NA, not applicable

^a^Adjusted for maternal baseline characteristics

In sensitivity analysis (Table 4), MZT were associated with lower odds of LGA (OR: 0.35; 95% CI, 0.18–0.64) and birth weight discordance (OR: 0.42; 95% CI, 0.19–0.83) as well as higher odds of PPROM (OR: 2.70; 95% CI, 1.53–4.81), preterm birth (OR: 3.42; 95% CI, 2.14–5.55), low birth weight (OR: 2.27; 95% CI, 1.43–3.65), and SGA (OR: 2.77; 95% CI, 1.29–6.07).

**Table 4:** Analysis of maternal and neonatal outcomes after PSM

Outcomes	DZT (*N* = 246)	MZT (*N* = 124)	OR (95% CI)
**Maternal outcomes**			
Hypertensive disorders in pregnancy	17 (6.91%)	7 (5.65%)	0.81 (0.30–1.92)
Gestational diabetes	18 (7.32%)	7 (5.65%)	0.76 (0.29–1.79)
Intrahepatic cholestasis of pregnancy	2 (0.81%)	2 (1.61%)	2.00 (0.24–16.8)
Placenta previa	3 (1.22%)	2 (1.61%)	1.33 (0.17–8.11)
Placental abruption	0 (0.00%)	0 (0.00%)	NA
Preterm premature rupture of the membranes	27 (11.0%)	31 (25.0%)	2.70 (1.53–4.81)
Cesarean delivery	241 (98.0%)	120 (96.8%)	0.62 (0.16–2.55)
**Neonatal outcomes**			
Neonatal death	1 (0.41%)	4 (3.25%)	8.24 (1.20–162)
Preterm birth, < 37 weeks	106 (43.3%)	86 (72.3%)	3.42 (2.14–5.55)
Very preterm birth, < 32 weeks	7 (2.86%)	4 (3.36%)	1.18 (0.30-4.00)
Extremely preterm birth, < 28 weeks	1 (0.41%)	1 (0.84%)	2.07 (0.08–52.6)
Low birth weight, < 2500 g	126 (51.4%)	84 (70.6%)	2.27 (1.43–3.65)
Very low birth weight, < 1500 g	9 (3.67%)	5 (4.20%)	1.15 (0.35–3.41)
Small for gestational age	13 (5.31%)	16 (13.4%)	2.77 (1.29–6.07)
Large for gestational age	64 (26.1%)	13 (10.9%)	0.35 (0.18–0.64)
Macrosomia, > 4000 g	1 (0.41%)	0 (0.00%)	NA
Birth weight discordance	44 (18.0%)	10 (8.4%)	0.42 (0.19–0.83)
Stillbirth	0 (0.00%)	1 (0.81%)	NA
Birth defects	7 (2.85%)	5 (4.07%)	1.45 (0.42–4.63)
Neonatal jaundice	3 (1.22%)	4 (3.36%)	NA
Pneumonia	0 (0.00%)	2 (1.68%)	NA
Respiratory distress syndrome	0 (0.00%)	0 (0.00%)	NA
Intestinal necrosis	0 (0.00%)	0 (0.00%)	NA

CI, confidence interval; DZT, dizygotic twins; MZT, monozygotic twins; NA, not applicable; OR, odds ratio

### Causal mediation analysis

Our analysis found that the association between MZT and neonatal death risk was partially mediated by preterm birth and low birth weight. The proportion of mediated effect by preterm birth was 20% (*P* < 0.05) using Model 2 variables and 15% (*P* < 0.05) using Model 3 variables. Similarly, the proportion mediated by low birth weight was 17% (*P* < 0.05) in Model 2 and 12% (*P* < 0.05) in Model 3. However, the proportion of the mediated effect of SGA to the total effect was not statistically significant, with a proportion of 3% (*P* > 0.05) in Model 2 and 2% (*P* > 0.05) in Model 3.

## Discussion

In this single-center retrospective study of 6101 DZT and 164 MZT births, MZT conceived by FET were associated with an increased risk of neonatal death as assessed by multivariate logistic regression models, double robust estimation, and Kaplan-Meier analysis, and the results were robust in sensitivity analysis.

ART-related MZT generally occur after the eight-cell stage of blastocyst [23–25], which contradicts the traditional belief that MZT arose from a split at the one-cell stage [26, 27]. Studies have shown that MZT are more common with blastocyst transfer than earlier embryo transfer [9, 14]. The higher rate of MZT after ART is believed to be due to various factors inherent to women undergoing fertility treatments and ART itself. These factors include maternal age, type of insemination, embryo developmental stage at embryo transfer, use of assisted hatching, embryo biopsy for preimplantation genetic testing, and FET [13, 14]. Numerous mechanisms have been proposed to explain ART-related MZT. These include disruptions in blastomere communication leading to the independent formation of two separate embryos, a breach or hardened zona from embryo manipulations techniques or culture conditions cutting the embryo in half, a thin or punctured zona causing the growing embryo to split or fall apart, a developmental delay that allows MZT occurrence, a possibility of trophoblast cells failing, collapsing, and regrowing at two sites [9, 14, 28–32]. Recent research has suggested that “overripe” ova or sperm may contribute, as these gametes are often frozen or stored for lengthy periods before use [33].

Prior research has indicated that many MZT pregnancies experience spontaneous reduction or loss before delivery due to factors such as twisted umbilical cords, major developmental anomalies, severe TTTS, and other vascular compromise [34, 35]. MZT pregnancies are also at a higher risk of stillbirth than the general population, with a 3-6-fold increase in the rate [36, 37]. In addition, MZT survivors are more likely to experience preterm delivery, intrauterine growth restrictions, birthweight discordance, maternal complications, and congenital anomalies [38–42]. Interestingly, although there may be a very slight overall increase in congenital anomalies in ART cases of MZT, it is not as high as the rates seen in spontaneous MZT [43, 44].

Consistent with previous literature, our study found that MZT were associated with an increased risk of adverse outcomes including PPROM, preterm birth, low birth weight, and SGA. While MZT had a slightly higher rate of stillbirth (1.22% vs. 0.80%), adjusted analyses revealed no statistically significant differences. Our study also revealed that MZT conceived through FET had an elevated risk of neonatal death, partially mediated by preterm birth and low birth weight. The proportion of mediated effect by preterm birth and low birth weight was relatively small, ranging from 12 to 20%, indicating that there might be other factors that contributed to the effect of MZT on neonatal death. These disadvantages of MZT are plausibly attributable to chorionicity, which confers an inferior perinatal and neonatal prognosis in monochorionic twins versus dichorionic twins [45, 46]. Cohort studies evaluating the clinical outcomes of twin pregnancies have shown that monochorionic twins have an increased risk of premature delivery, lower birth weight, intertwin birth weight discordance, stillbirth, major neonatal morbidity, and neonatal mortality compared to dichorionic twins [16–19]. Additionally, ART appears to raise the already high perinatal risks of monochorionic twins compared to natural conception [17]. Gestational comorbidities, such as gestational diabetes, gestational hypertension, were also more common in monochorionic pregnancies [47]. Our findings have important implications for clinicians and policymakers involved in the care of MZT conceived through ART. Close monitoring of MZT pregnancies should be conducted, and strategies to prevent preterm birth and low birth weight should be implemented to reduce the risk of neonatal death. Comprehensive counseling should be provided for patients considering ART, including the risks associated with MZT. Further research is also needed to understand the underlying mechanisms that lead to MZT conceived through ART and identify other potential factors that may contribute to the increased risk of neonatal death in MZT.

Almost all of the studies defined MZT as two fetal poles within a single gestational sac or an exceeding number of fetal poles compared to the embryos transferred. This definition, however, excludes DCDA MZT and thus does not accurately reflect the incidence of MZT. The reports pertaining to MZT, upon closer inspection, were found to be reports of MCDA MZT. To obtain a more accurate assessment of MZT incidence, studies based on single embryo transfer should be considered, considering the possibility of concurrent natural conceptions [48]. Genetic testing would be a more accurate method albeit with increased invasiveness, costs, and potential reduction of the sample size [49]. The accurate definition and identification of MZT persist as a significant obstacle that necessitates attention. An optimal study design would entail the utilization of DNA testing and embryo time-lapse examination to discern potential indications of embryo splitting.

There are certain limitations to this study. Firstly, the retrospective design of the study may have introduced bias. Secondly, the determination of MZT arising from both single and multiple embryo transfers based on MCDA placentation in ultrasound analysis may lead to erroneous classification of DCDA MZT as DZT. Meanwhile, this definition cannot exclude MCDA DZT, a rare but possible event. However, due to the large number of DCDA DZT cases and the rarity of DCDA MZT or MCDA DZT events, it can be reasonably inferred that these events display a limited confounding effect on our findings. Moreover, these occurrences are expected to dilute detected associations rather than inflate them. Thirdly, this study may only capture strong associations due to the rare event and limited sample size. Fourthly, the available data were not able to identify which twin had adverse outcomes. Lastly, our findings could still be influenced by some unmeasured confounders despite the application of advanced epidemiological methods. Nevertheless, there are certain strengths to this study needs to be acknowledged. It included a relatively large population of females with twins born after FET. The inclusion criteria allowed for maximal capture of MZT. And several association inference models were applied to evaluate the credibility of the study results.

## Conclusions

This paper provides a comprehensive comparison of maternal and perinatal outcomes between MZT and DZT conceived through FET. The evidence suggests that MZT are related to an increased risk of neonatal death. Therefore, at-risk ART pregnancies involving MZT require thorough clinical examinations, prognostic assessments, and antenatal monitoring to ensure optimal outcomes.

## Data Availability

All of the study’s data are included in the publication.
